# The complete mitochondrial genome of *Rhynchocypris oxycephalus* (Teleostei: Cyprinidae) and its phylogenetic implications

**DOI:** 10.1002/ece3.5369

**Published:** 2019-06-20

**Authors:** Zhichao Zhang, Qiqun Cheng, Yushuang Ge

**Affiliations:** ^1^ Key Laboratory of Oceanic and Polar Fisheries, Ministry of Agriculture and Rural Affairs, East China Sea Fisheries Research Institute Chinese Academy of Fishery Sciences Shanghai China; ^2^ Wuxi Fisheries College Nanjing Agricultural University Wuxi China; ^3^ College of Marine Sciences Shanghai Ocean University Shanghai China

**Keywords:** conservation genetics, DNA barcoding, mitochondrial genome, phylogenetic analysis, *Rhynchocypris oxycephalus*

## Abstract

*Rhynchocypris oxycephalus* (Teleostei: Cyprinidae) is a typical small cold water fish, which is distributed widely and mainly inhabits in East Asia. Here, we sequenced and determined the complete mitochondrial genome of *R. oxycephalus* and studied its phylogenetic implication. *R. oxycephalus* mitogenome is 16,609 bp in length (GenBank accession no.: MH885043), and it contains 13 protein‐coding genes (PCGs), two rRNA genes, 22 tRNA genes, and two noncoding regions (the control region and the putative origin of light‐strand replication). 12 PCGs started with ATG, while COI used GTG as the start codon. The secondary structure of tRNA‐Ser (AGN) lacks the dihydrouracil (DHU) arm. The control region is 943bp in length, with a termination‐associated sequence, six conserved sequence blocks (CSB‐1, CSB‐2, CSB‐3, CSB‐D, CSB‐E, CSB‐F), and a repetitive sequence. Phylogenetic analysis was performed with maximum likelihood and Bayesian methods based on the concatenated nucleotide sequence of 13 PCGs and the complete sequence without control region, and the result revealed that the relationship between *R. oxycephalus* and *R. percnurus* is closest, while the relationship with *R. kumgangensis* is farthest. The genus *Rhynchocypris* is revealed as a polyphyletic group, and *R. kumgangensis* had distant relationship with other *Rhynchocypris* species. In addition, COI and ND2 genes are considered as the fittest DNA barcoding gene in genus *Rhynchocypris*. This work provides additional molecular information for studying *R. oxycephalus* conservation genetics and evolutionary relationships.

## 1 INTRODUCTION

**Figure 1:**
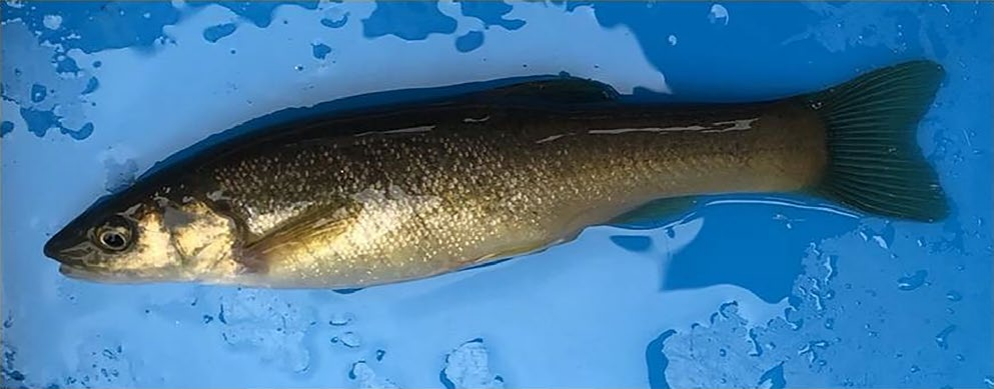
Photograph of *Rhynchocypris oxycephalus*

*Rhynchocypris oxycephalus* (Cyprinidae, Cypriniformes, and Osteoichthyes) is a small cold water species (Figure 1), which generally inhabits higher altitudes and lower water temperatures, high dissolved oxygen, upstream of the stream or mountain stream with sand or stone (Liang, Sui, Chen, Jia, & He, 2014; Zhang et al., 2011). It is an omnivorous fish that usually feeds on invertebrates, aquatic insect larvae, or plant debris. *R. oxycephalus* often has a large population, acting as the dominant species and playing a crucial role in maintaining the balance of stream ecosystem (Park, Im, Ryu, Nam, & Dong, 2010). Due to poor diffusion ability, *R. oxycephalus* is an ideal materials for the study of freshwater fish biogeography.

The phylogenetic relationship of genus *Rhynchocypris* was very complicated, and it is one of the long‐standing controversial scientific issues in the classification of the subfamily Leuciscinae. Formerly, genus *Rhynchocypris* was considered as synonym with genus *Phoxinus* (Nelson & Joseph, 1976). Based on isozyme, Ito, Sakai, Shedko, and Jeon (2002) found that genus *Phoxinus* and genus *Rhynchocypris* were two nature taxa with close relationship. Based on 16S rRNA and Cytb genes from the mitochondrial genome, Sasaki et al. (2007) found that relationship between genus *Phoxinus* and genus *Rhynchocypris* is a little farther and *Rhynchocypris* was sister group with genus *Tribolodon* and genus *Pseudaspius*. In above studies, phylogenetic relationship of genus *Rhynchocypris* is controversial and further research is needed.

The typical vertebrate mitochondrial genome is circular, ranging in size from ~15 to 18 kb and generally containing 37 genes (13 protein‐coding genes, 22 tRNAs, and two rRNAs) and two noncoding regions (control region and putative origin of light‐strand replication; Sasaki et al., 2007). Because of its maternal inheritance, high mutation rate, and small molecular weight, mitochondrial DNA has been used as a good molecular marker in phylogenetic analysis.

In addition, the mitochondrial gene fragments have different evolution rates, so different gene fragments can be applied to different species studies. For example, RNA has a slower evolution rate and relatively conservative genes, which is suitable for species research in the upper class. ND, COXI, and other genes are faster than RNA genes in rate of evolution, and they are suitable for phylogenetic analysis between species or genus.

Due to the limitations of morphological classification methods, more and more molecular biology methods have been applied to fish species identification in recent years. DNA barcoding technology is the most widely used among them (Hogg & Hebert, 2004). DNA barcoding technology is a technique for rapidly identifying species by analyzing the DNA sequences of standard target genes. It can not only identify known species, but also discover new species and hidden species that cannot be identified by traditional taxonomic methods. Compared with traditional species identification methods, this technology has the advantages of high accuracy, high efficiency, and is not affected by the environment of the identified object, individual factors of individual development, and identification experts (Hebert, Ratnasingham, & Dewaard, 2003). In mitochondrial genomes, COI gene is commonly used for species identification of birds (Yoo et al., 2013), insects (Hajibabaei, Janzen, Burns, Hallwachs, & Hebert, 2006), and fishes (Ward, Zemlak, Innes, Last, & Hebert, 2005) and has achieved good effect. However, as DNA barcoding, COI gene is not suitable for all animal species. For example, Li, Liu, Li, Du, and Zhuang (2015) analyzed Clupeiformes with COI gene and found that although all species can be distinguished, the efficiency is ordinary. Under this situation, more mitochondrial genes should be used as animal DNA barcodings. For example Miya and Nishida (2000a, 2000b), Zardoya and Meyer (1996), and Chen, Chi, Mu, Liu, and Zhou (2008) considered that COI, COIII, ND2, ND4, ND5, and Cytb genes were the best molecular markers for phylogenetic analysis in the research of Vertebrate and Teleostean. So these genes have the potential to be good DNA barcodings.

Relative to a mitochondrial gene fragment, the complete mitochondrial genome has complete mitochondrial genetic information with a large amount of information. It can reveal the evolution of mitochondrial molecules more comprehensively. In the classification of species, phylogenetic relationship based on the complete sequence of mitochondria can be used as a reference.

In this study, we designed primers for the amplification of the full sequence of mitochondrial genome, which can also be used as references to amplify the full mitochondrial genome of other Cyprinid fishes, and determined the complete mitochondrial genome of *R. oxycephalus*. In addition, we described genome organization, gene arrangement, and characterization of *R. oxycephalus*. On this basis, we analyzed the complete mitochondrial genome of *R. oxycephalus* and aligned the sequence with other species to explore its phylogenetic relationship in *Rhynchocypris* and *Leuciscus*. Moreover, we aimed to find the effective DNA barcoding among *Rhynchocypris* species to facilitate the identification of *Rhynchocypris* species.

## 2 MATERIALS AND METHODS

### 2.1 Sample collection and DNA extraction

Individuals of *R. oxycephalus* were collected from Qingyang County, Anhui province, China, in May 2018. The muscle was preserved in 95% ethanol and stored at −20°C until DNA extraction was performed. Genomic DNA was extracted from the muscle using the Column mtDNA out kit (Sangon, Shanghai, China) and stored at −20°C until needed for PCR.

### 2.2 PCR amplification and sequencing

PCR primers were designed by Primer Premier 5.0 software (Lalitha, 2000) and were based on universal primers of fish mtDNA (Simon et al., 1994). In addition, we used 16 sets of specific primers to amplify overlapping segments of the complete mitochondrial genome in *R. oxycephalus*. The specific primers were designed based on the alignments of the relatively conserved regions of *Phoxinus oxycephalus* (GenBank accession nos.: NC_027273; Sui, Liang, & He, 2016 and NC_018818; Imoto et al., 2013), and the specific primer sequences are shown in Table 1. PCR amplification was performed in a 20 μl reaction volume containing about 10 μl Premix Taq, 1 μl template, 7 μl ddH_2_O, and 1 μl each primer. The amplification condition was an initial denaturation for 5 min at 95°C, followed by 35 cycles of denaturation for 30 s at 94°C, annealing for 30 s at 50–55°C, then extension at 72°C for 1 min followed by a final extension at 72°C for 10 min. PCR products were separated by 1.0% agarose gel electrophoresis. All PCR fragments were sequenced after separation and purification at Map Biotech Inc (Shanghai, China).

**Table 1:** The 16 primer combinations for amplifying the complete mitochondrial DNA of *Rhynchocypris oxycephalus*

Primer name	Primer sequence(5'–3')
Rhynchocypris‐1F	GACGAGGAGCGGGCATCAGG
Rhynchocypris‐1R	CGGGGTATCAAACTAAAGGTC
Rhynchocypris‐2F	CCAACACCACAAACTAAACCAT
Rhynchocypris‐2R	TCTAGCCATTCATACAGGTCTCT
Rhynchocypris‐3F	CAACGAACCAAGTTACCCAAG
Rhynchocypris‐3R	GTGCCCAAAAATAGTACGACTG
Rhynchocypris‐4newF	AACCTGTTCGCCCCTCTACCT
Rhynchocypris‐4newR	GGCAAGGAAGGCTGCGGATGT
Rhynchocypris‐5F	CCTCTTAACGGCCTTTGGACT
Rhynchocypris‐5R	TTCCAAACCCTCCAATAAGAA
Rhynchocypris‐6F	GTGACAGCCGTCCTTCTCCTC
Rhynchocypris‐6R	GTAAGTTTGGTTGAGACTATCGC
Rhynchocypris‐7NEWF	ACCCCTGTATGTCTTGAGCTC
Rhynchocypris‐7NEWR	ATTAGTTGATTGGTAAATCGGTTC
Rhynchocypris‐8F	ATAARACTGACTCCTGAACCTGA
Rhynchocypris‐8R	GCCTGGAGAGCGGTAAAATAA
Rhynchocypris‐9F	AGGAGTTATTACGCTGGACCC
Rhynchocypris‐9R	GTTRAGGTTTTGTAGGCGGTC
Rhynchocypris‐10NEWF	GGTTAGCATTTCATCGCACACA
Rhynchocypris‐10NEWR	TGGGTTCGTTCATAGGCTGT
Rhynchocypris‐11F	TGCCTACGACAAACAGACCTTA
Rhynchocypris‐11R	GTGTAATCATGGCTACCAAGAA
Rhynchocypris‐12F	GCGTTCGACACAAACATTAGCT
Rhynchocypris‐12R	AATGGATTGTCCTCGCTGAT
Rhynchocypris‐13F	TRGCACTGACAGGCACCCCAT
Rhynchocypris‐13R	GTTYTAATTGTGGGTTTAATTGCT
Rhynchocypris‐14F	AAAGRACGAGGGATAAGAAGGA
Rhynchocypris‐14R	CCCTGTCTCGTGTAGAAAGAGCA
Rhynchocypris‐15F	AGACCTCCTTGGCTTTGTAGTA
Rhynchocypris‐15R	TGTTGGGTAACGAGGAGTATG
Rhynchocypris‐16F	ATGATAGAACCAGGGACACAAT
Rhynchocypris‐16NEWR	TATTGCTCCTCCTAACCACCC

### 2.3 Sequencing assembling and annotation

The complete mitochondrial genome sequences were assembled and annotated with the software Geneious (Drummond et al., 2010). Locations of PCGs and rRNA genes were annotated by comparisons with genes From *Phoxinus oxycephalus* (NC_027273, NC_018818). PCG boundaries were identified by ORF Finder (http://www.ncbi.nlm.nih.gov/gorf/gorf.html). The tRNA genes were identified using the Online Program tRNAscan‐SE 2.0 (http://lowelab.ucsc.edu/tRNAscan-SE/; Lowe & Chan, 2016) and used the software RNAstructure 5.6 (Reuter & Mathews, 2010) to predict the secondary structure of mitochondrial tRNA. The putative tRNAs that were not found by these two tools were identified based on sequence similarity to tRNAs of the other previously published Cyprinidae mitochondrial genome. The secondary structure of the putative origin of light‐strand replication was analyzed with the software RNAstructure 5.6 (Reuter & Mathews, 2010). Nucleotide composition and codon usage were analyzed by Mega 5.0 (Tamura et al., 2011). Composition skew analysis was carried out with the formula AT‐skew = [A − T]/[A + T] and GC‐skew = [G − C]/[G + C], respectively (Nicole & Thomas, 1995). The tandem repeats of putative control regions were analyzed with the Tandem Repeats Finder program (http://tandem.bu.edu/trf/trf.advanced.submit.html; Benson, 1999). The gene map of the *R. oxycephalus* mitochondrial genome was drawn by the online software Ogdraw (https://chlorobox.mpimp-golm.mpg.de/OGDraw.html; Lohse, Drechsel, Kahlau, & Bock, 2013).

### 2.4 Sequence alignment and phylogenetic analysis

Sequence alignment and phylogenetic analyses of *Rhynchocypris* species were performed with 8 complete mitochondrial genomes of the *Rhynchocypris* species downloaded from GenBank. Multiple alignments of the mitochondrial gene sequences were used by Clustal X 1.83 (Jeanmougin, Thompson, Gouy, Higgins, & Gibson, 1998) with the default settings. Length of consensus sequences, amount of variable sites, Kimura 2‐Parameter (K2P) distance, and Ts/Tv ratios were calculated by the software Mega5.0 (Tamura et al., 2011). The Online Program Gblock 0.91b (http://www.phylogeny.fr/one_task.cgi?task_type=gblocks) with default settings was used to find the conserved regions of the sequence (Castresana, 2000). Before the establishment of phylogenetic tree, the substitution saturation of base was tested by DAMBE software with GTR distance (Xia, 2013). For likelihood ratio tests, Modeltest 3.7 (Posada & Crandall, 1998) and Akaike information criterion (AIC; Bozdogan, 1987) were used to determine the best‐fitting model of the analysis. Maximum likelihood (ML) analysis of the 13 PCGs in 8 species of *Rhynchocypris* fish was also used by Mega 5.0 (Tamura et al., 2011), with *Acrossocheilus fasciatus* used as outgroups. The support values of the ML tree were evaluated via a bootstrap test with 1,000 iterations. In this analysis, “GTR + G” model was considered as the best‐fit model.

Further, to explore the evolutionary relationships within *Leuciscus*, the complete mitochondrial genome of seven other *Leuciscus* fish species was downloaded from the Genbank. Maximum likelihood analysis of the complete mitochondrion genome among *Leuciscus* fishes was performed using Mega 5.0 (Tamura et al., 2011), with *Acrossocheilus fasciatus* as outgroup. Bayesian (BI) analysis was carried out using MrBayes v.3.2.6 (Huelsenbeck & Ronquist, 2005). Bayesian posterior probabilities were estimated using the Markov chain Monte Carlo (MCMC) sampling approach. Bayesian analysis starts with a random tree, runs 4 Markov chains at the same time, samples once every 100 generations, removes 25% of the aging samples that start running, and builds a consistent tree with the remaining samples. The control region was removed in both analysis due to its large variability. For ML and BI analysis, an optimum model of GTR + I + G (nst = 6; rates = gamma) was selected by AIC in Modeltest 3.7 (Posada & Crandall, 1998).

### 2.5 The analysis of DNA barcoding

Six PCGs (COI, COIII, ND2, ND4, ND5, and Cytb) were selected as potential DNA barcoding of *Rhynchocypris* species to find the fittest one. We downloaded other 15 complete mitochondrial genome of *Rhynchocypris* species from Genbank including two sequences of *Phoxinus oxycephalus* (NC_027273; Sui et al., 2016, KP641342; Sui et al., 2016), two sequences of *R. percnurus* (AP009061; Imoto et al., 2013, NC_015360; Imoto et al., 2013), four sequences of *R. lagowskii* (KJ641843; Sun, Wang, & Wei, 2016, AP009147; Imoto et al., 2013, NC_015354; Imoto et al., 2013, KR091310; Unpublished), one sequence of *R. percnurus mantschuricus* (NC_008684; Saitoh et al., 2006), one sequence of *R. p. sachalinensis* (NC_015362; Imoto et al., 2013), two sequences of *R. kumgangensis* (NC_019614; Yun, Yu, Kim, & Kwak, 2012, AP011363; Unpublished), one sequence of *R. semotilus* (NC_029341; Imoto et al., 2013), and two sequences of *R. oxycephalus jouyis* (NC_018818; Imoto et al., 2013, AP011269; Miya et al., 2015). The variation rate, K2P interspecies, and intraspecies distances were calculated by Mega 5.0 (Tamura et al., 2011). Based on the K2P interspecies and intraspecies distances, Wilcoxon signed rank test was conducted in the software SPSS 19.0 (Field, 2013) to compare the differences in 6 PCGs.

## 3 RESULTS

### 3.1 Genome annotation and base composition

**Figure 2:**
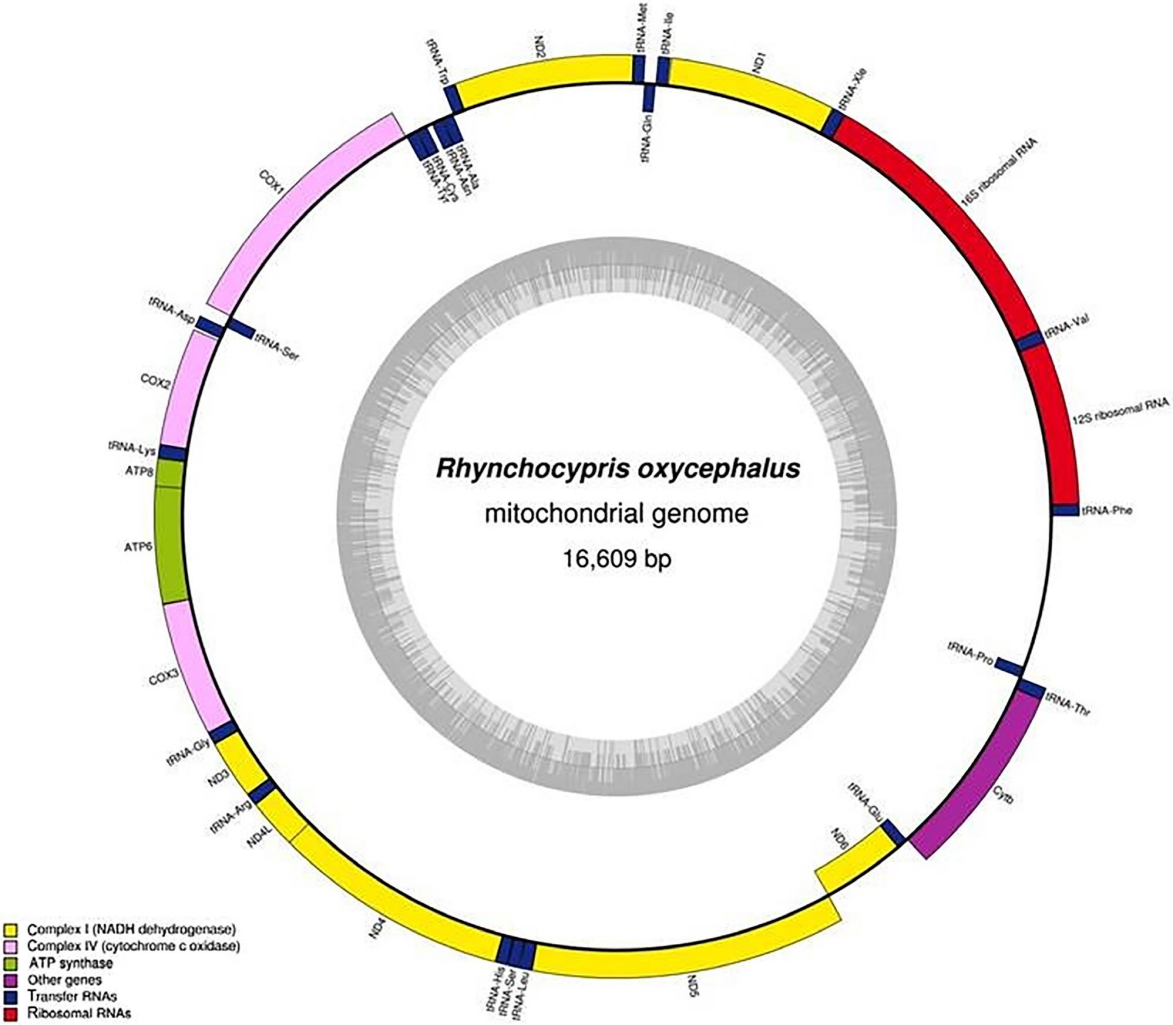
Gene map of the *Rhynchocypris oxycephalus* mitochondrial genome

We obtained the mitochondrial genome sequence of *R. oxycephalus* and deposited it in NCBI with GenBank accession no. MH885043. The mitogenome of *R. oxycephalus* was a circular DNA molecule with 16,609 bp in length. As shown in Figure 2, the mitogenome organization of *R. oxycephalus* was similar to that of typical vertebrate mitochondrial genome, it contained 13 PCGs, 22 transfer RNA genes, 2 ribosomal RNAs, and 2 noncoding regions. The basic information and genomic structure of the gene sequences are shown in the Table 2. The mitogenome structure of *R. oxycephalus* showed that its position was consistent with most Cyprinidae fishes (Zhang, Yue, Jiang, & Song, 2009). The light chain (L chain) encoded only the ND6 gene and 8 tRNA genes (tRNA‐Gln, tRNA‐Trp, tRNA‐Ala, tRNA‐Asn, tRNA‐Cys, tRNA‐Ser, tRNA‐Glu, and tRNA‐Pro). Most mitochondrial genes were encoded on the heavy chain (H chain).

**Table 2:** Characteristics of the mitochondrial genome of *Rhynchocypris oxycephalus*

Feature	Length/bp	Position	Start codon	Stop codon	Anticodon	Intergenic nucleotide	Number of amino acid	Strand
tRNA^Phe^	69	1–69			GAA	0		H
12S rRNA	957	70–1026				0		H
tRNA^Val^	72	1,027–1098			TAC	0		H
16S rRNA	1690	1,099–2788				0		H
tRNA^Leu^	76	2,789–2864			TAA	1		H
ND1	975	2,866–3840	ATG	TAA		4	324	H
tRNAIle	72	3,845–3916			GAT	−2		H
tRNA^Gln^	71	3,915–3985			TTG	1		L
tRNA^Met^	69	3,987–4055			CAT	0		H
ND2	1,045	4,056–5100	ATG	T		0	348	H
tRNA^Trp^	71	5,101–5171			TCA	1		H
tRNA^Ala^	69	5,173–5241			TGC	1		L
tRNA^Asn^	73	5,243–5315			GTT	0		L
OL	36	5,316–5351				−3		L
tRNA^Cys^	68	5,349–5416			GCA	1		L
tRNA^Tyr^	71	5,418–5488			GTA	1		L
COI	1551	5,490–7040	GTG	TAA		0	516	H
tRNA^Ser^	71	7,041–7111			TGA	3		L
tRNA^Asp^	74	7,115–7188			GTC	13		H
COII	691	7,202–7892	ATG	T		0	230	H
tRNA^Lys^	76	7,893–7968			TTT	1		H
ATP8	165	7,970–8134	ATG	TAG		−7	54	H
ATP6	684	8,128–8811	ATG	TAA		−1	227	H
COIII	784	8,811–9594	ATG	T		0	261	H
tRNA^Gly^	71	9,595–9665			TCC	0		H
ND3	349	9,666–10014	ATG	T		0	116	H
tRNA^Arg^	69	10,015–10083			TCG	0		H
ND4L	297	10,084–10380	ATG	TAA		0	98	H
ND4	1,382	10,374–11755	ATG	TA		−7	460	H
tRNA^His^	69	11,756–11824			GTG	0		H
tRNa^Ser^	68	11,825–11892			GTC	0		H
tRNA^Leu^	73	11,894–11966			TAG	1		H
ND5	1836	11,967–13802	ATG	TAA		0	611	H
ND6	522	13,799–14320	ATG	TAA		−4	173	L
tRNA^Glu^	68	14,321–14388			TTC	0		L
Cytb	1,141	14,391–15531	ATG	T		2	380	H
tRNA^Thr^	72	15,532–15603			TGT	0		H
tRNA^Pro^	71	15,603–15673			TGG	−1		L
D‐loop	936	15,674–16609				0		H

The total base composition of *R. oxycephalus* mitochondrial genome was A:28.7%, T:27.3%, C:26.2%, G:17.8%, and exhibited positive AT‐skew (0.030) and GC‐skew (0.196), which was consistent with the lowest frequency for G content in typical fishes’ mitochondrial genomes (Perna & Kocher, 1995). The overall A + T content of the mitochondrial genome of *R. oxycephalus* was 56.0%; such an A‐T‐rich pattern reflected the typical sequence feature of the vertebrate mitochondrial genome (Mayfield & Mckenna, 1978).

The *R. oxycephalus* mitochondrial genome contained 25 overlapping nucleotides. These were located in 7 pairs of neighboring genes and varied in length from 1 to 7 bp; one of the longest overlap (7 bp) was located between ND4L and ND4, the other was located between ATP8 and ATP6. A total of 30 intergenic nucleotides were dispersed in 12 locations and ranged in size from 1 to 13 bp; the longest intergenic spacer (13 bp) was located between tRNA‐Asp and COII.

### 3.2 Protein‐coding genes

**Figure 3:**
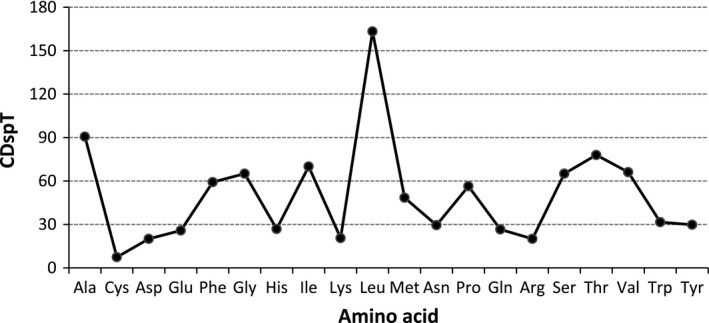
Codon distribution in *Rhynchocypris oxycephalus*. CDspT‐codons per thousand codons

Among 13 PCGs of *R. oxycephalus*, there were 12 PCGs using ATG as the initiation codon except the COI gene, which used GTG as initiation codon. All COI genes in reported fishes used GTG as initiation codon. Thus, the feature that COI used GTG as initiation codon seemed to be prevalent among nontetrapod vertebrates (Saitoh et al., 2000). However, stop codons varied among 13 PCGs. Seven PCGs in *R. oxycephalus* mitochondrial genome ended with complete stop codons, including TAA (ND1, COI, ATP6, ND4L, ND5, and ND6) and TAG (ATP8), the rest six genes ended with incomplete stop codons, either TA (ND4) or T (ND2, COII, ND3, COIII, and Cytb), which were presumably completed as TAA after transcriptions (Anderson et al., 1981). The codon usage and the relative synonymous codon usage (RSCU) in *R. oxycephalus* mitochondrial genome are given in Table 3. It revealed that codons were abundant in A or T in third position. The codons that had relatively high content of G and C were likely to be abandoned. Codon distribution in *R. oxycephalus* is given in Figure 3. Codons per thousand codons (CDspT) of *R. oxycephalus* showed its preference to Leucine and Alanine.

**Table 3:** Codon usage in *Rhynchocypris oxycephalus* mitochondrial protein‐coding genes

Codon	Count	RSCU	Codon	Count	RSCU	Codon	Count	RSCU	Codon	Count	RSCU
**UUU(F)**	**113**	**1**	UCU(S)	44	1.07	**UAU(Y)**	**76**	**1.35**	UGU(C)	14	1
UUC(F)	112	1	UCC(S)	58	1.41	UAC(Y)	37	0.65	UGC(C)	14	1
UUA(L)	137	1.33	**UCA(S)**	**73**	**1.78**	UAA(*)	6	3.43	**UGA(W)**	**95**	**1.58**
UUG(L)	31	0.3	UCG(S)	13	0.32	UAG(*)	1	0.57	UGG(W)	25	0.42
CUU(L)	142	1.37	CCU(P)	43	0.8	CAU(H)	38	0.75	CGU(R)	15	0.79
CUC(L)	88	0.85	CCC(P)	70	1.31	**CAC(H)**	**64**	**1.25**	CGC(R)	16	0.84
**CUA(L)**	**168**	**1.63**	**CCA(P)**	**77**	**1.44**	**CAA(Q)**	**84**	**1.66**	**CGA(R)**	**37**	**1.95**
CUG(L)	54	0.52	CCG(P)	24	0.45	CAG(Q)	17	0.34	CGG(R)	8	0.42
**AUU(I)**	**188**	**1.41**	ACU(T)	41	0.55	**AAU(N)**	**62**	**1.11**	AGU(S)	14	0.34
AUC(I)	78	0.59	ACC(T)	111	1.5	AAC(N)	50	0.89	**AGC(S)**	**44**	**1.07**
**AUA(M)**	**124**	**1.36**	**ACA(T)**	**121**	**1.64**	**AAA(K)**	**59**	**1.51**	AGA(*)	0	0
AUG(M)	59	0.64	ACG(T)	23	0.31	AAG(K)	19	0.49	AGG(*)	0	0
GUU(V)	79	1.25	GCU(A)	62	0.72	GAU(D)	27	0.7	GGU(G)	34	0.55
GUC(V)	38	0.6	**GCC(A)**	**145**	**1.69**	**GAC(D)**	**50**	**1.3**	GGC(G)	71	1.15
**GUA(V)**	**96**	**1.52**	GCA(A)	109	1.27	**GAA(E)**	**71**	**1.45**	**GGA(G)**	**84**	**1.36**
GUG(V)	39	0.62	GCG(A)	28	0.33	GAG(E)	27	0.55	GGG(G)	58	0.94

* Stop codons; the letters in brackets are abbreviations of each amino acid, preferred codons in bold.

### 3.3 Ribosomal and transfer RNA genes

The 12S and 16S rRNA genes of *R. oxycephalus* mitochondrion were 957 and 1693 bp in length, respectively. As in other vertebrates, they were located between tRNA‐Phe and tRNA‐Leu (UUR) genes and separated by tRNA‐Val gene. The base composition of the two rRNA gene sequences was A: 28.6%, T:26.6%, C:21.1%, and G:23.7%. The A + T and G + C contents of the two rRNA were found to be 53.4% and 46.6%, respectively.

**Figure 4:**
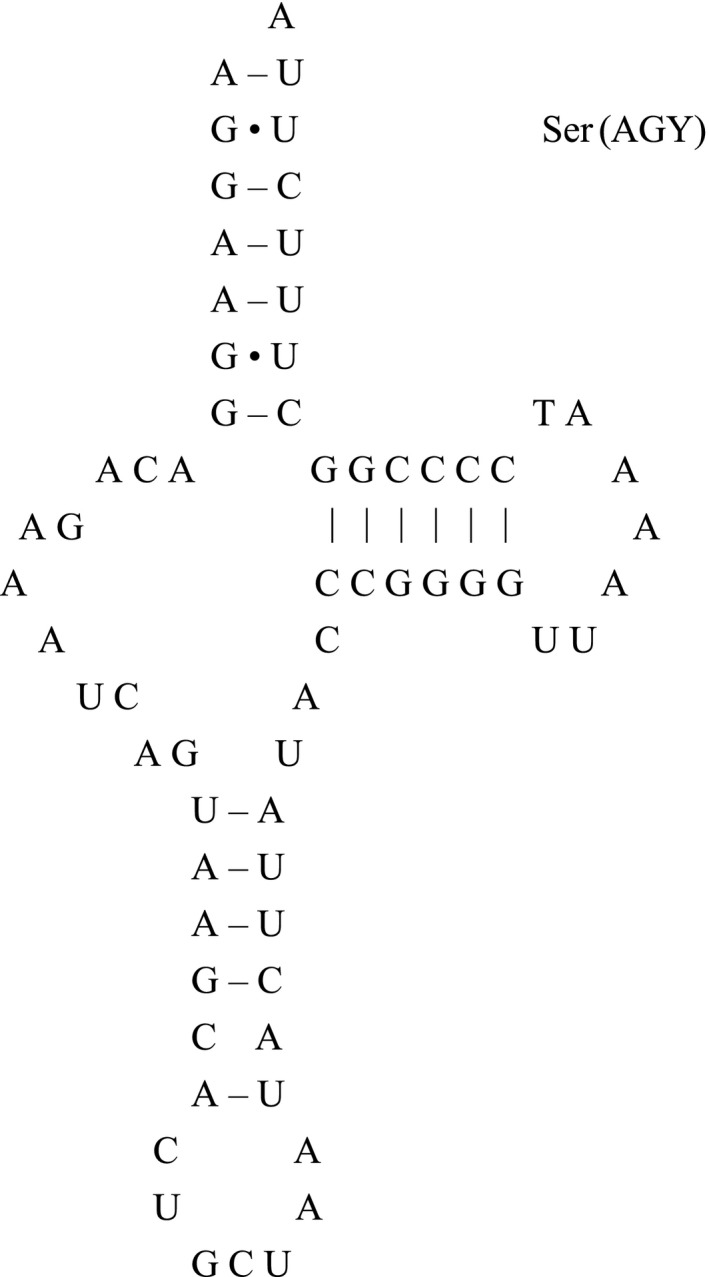
The secondary structures of the tRNA‐Ser(AGY) genes in* Rhynchocypris oxycephalus*

The secondary structure of the animal tRNA gene was very similar. It showed a typical clover stem‐loop structure including four arms and four rings, one of which was a variable ring. According to its function, the four arms and the ring were, respectively named: amino acid accepting arm, dihydrouracil arm (DHU) and loop, anticodon arm and loop, TψC arm and loop, and a variable loop. The mitochondrial genome of *R. oxycephalus* contained 22 tRNAs, 14 of which were located on the heavy chain H chain and 8 are located on the light chain L chain with a gene length of 68–76 bp. The average base composition of 22 tRNAs was found to be A: 32.9%, G: 22.3%, T: 20.5%, C: 24.3%. All the tRNA genes included two tRNA‐Ser and tRNA‐Leu, while the other had only one. Among the 22 tRNA genes, tRNA‐Ser (AGY) lacks a DHU arm (Figure 4), and the rest were typical clover structures, accompanied by UU, AA, and GU mismatches. Compared with other *Rhynchocypris* species, most of the mismatched nucleotides were G‐U pairs, which could form a weak bond in tRNAs and noncanonical pairs in tRNA secondary structures (Gutell, Lee, & Cannone, 2002).

### 3.4 Noncoding regions

Like other vertebrates, there were two noncoding regions in *R. oxycephalus* mitochondrial genome. One was control region (D‐loop), and the other was putative origin of light‐strand replication (O_L_).

Control region of *R. oxycephalus* mitochondrial genome was 943 bp in length, locating between tRNA‐Pro and tRNA‐Phe genes. It was also called A + T‐rich region with A + T content accounting for 65% of total base pairs, which was much higher than G + C content. Similar result was observed in other Cyprinidae species (Zhang et al., 2009).

**Figure 5:**
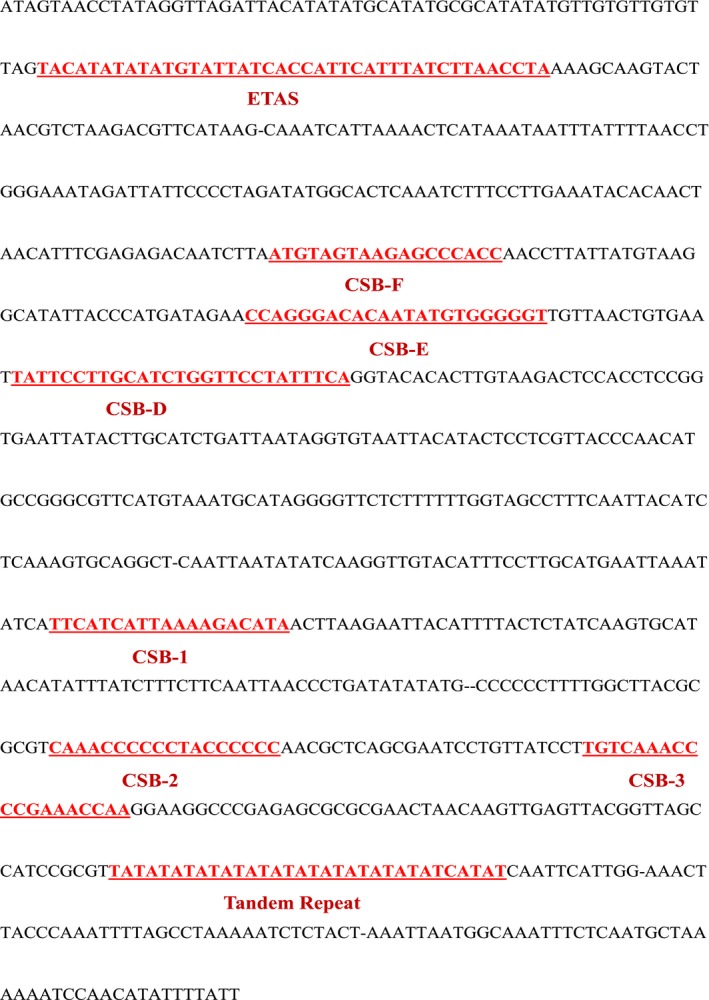
Schematic map characterizing of the control region of *Rhynchocypris oxycephalus*. ETAS‐extended termination‐associated sequence, CSB‐conserved sequence blocks

Control region consisted of termination‐associated sequence (TAS), central conserved domain (CCD), and conserved sequence block (CSB). TAS had an obvious hairpin structure (TACAT and ATGTA; Guo, Liu, & Liu, 2003). Liu (2002) identified three conserved sequence blocks (CSB‐D, CSB‐E, and CSB‐F) from CCD. In addition, previous studies on mammalian conserved sequence regions had found that there were generally three conserved sequences in CSB, which were named CSB1, CSB2, and CSB3, and speculated that this region was involved in heavy chain RNA primer generation (Walberg & Clayton, 1981). In addition, one repetitive sequence (AT) was found by the software Tandem Repeat Finder. This repetitive sequence was also found in other Cyprinidae species (Liu, 2002). By comparing with the nucleotide sequences of other Cyprinidae fishes, all sequences features were found (ETAS: 5′‐TACATATATATGTATTATCACCATTCATTTATCTTAACCTA‐3′; CSB‐F:5′‐ATGTAGTAAGAGCCCACC‐3′; CSB‐E: 5′‐CCAGGGACACAATATGTGGGGGT‐3′; CSB‐D: 5′‐TATTCCTTGCATCTGGTTCCTATTTCA‐3′; CSB‐1: 5′‐TTCATCATTAAAAGACATA‐3′; CSB‐2: 5′‐CAAACCCCCCTACCCCCC‐3′; CSB‐3: 5′‐TGTCAAACCCCGAAACCAA‐3′). All sequences features of *R. oxycephalus* in control region are shown as Figure 5.

**Figure 6:**
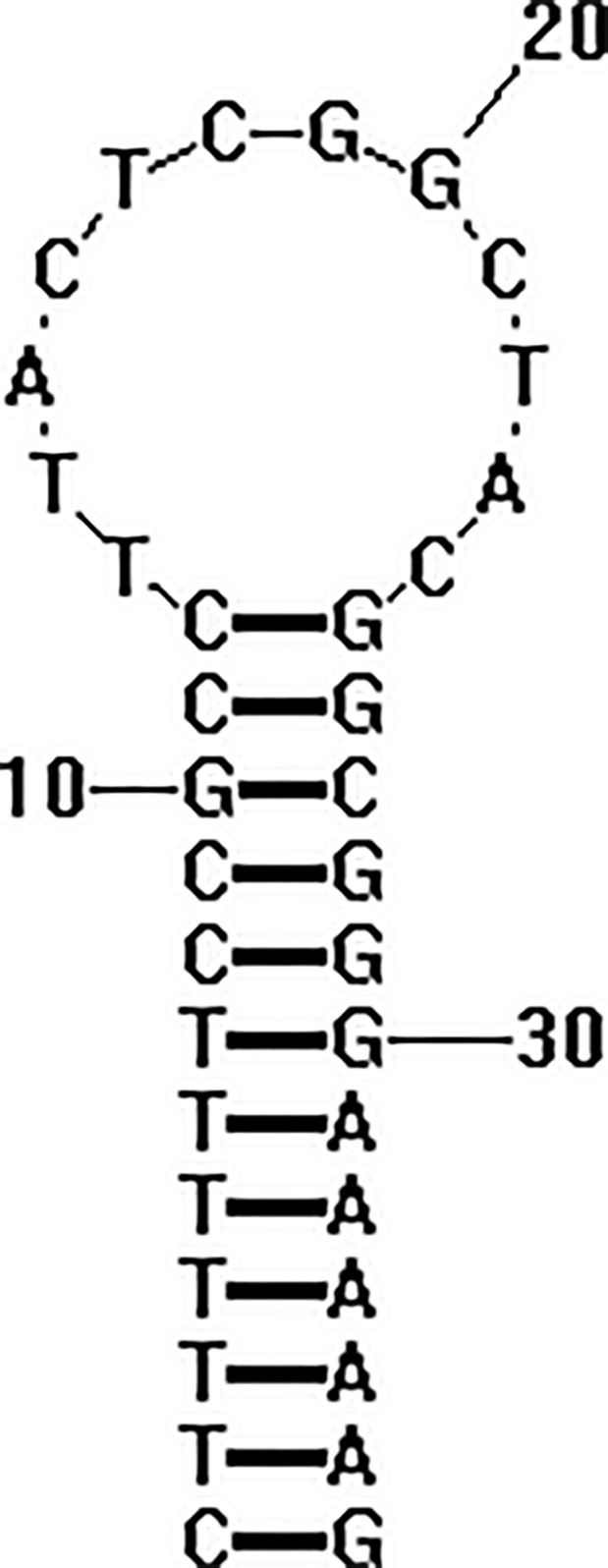
The secondary structures of the putative origin of light‐strand replication gene in* Rhynchocypris oxycephalus*

Putative origin of light‐strand replication (O_L_) was located in a cluster of five tRNA genes (WANCY region) between tRNA‐Asn and tRNA‐Cys which was similar to other vertebrates. The length of the putative origin of light‐strand replication was 36 bp. The region could fold into a stable stem‐loop secondary structure which included 12 bp loop area and 24 bp stem area. The stem‐loop structure was generally a characteristic of the origin of light‐strand replication, and it was closely related to the replication of mitochondrial DNA (Kawaguchi, Miya, & Nishida, 2001). The secondary structure of the putative origin of light‐strand replication in *R. oxycephalus* is shown in Figure 6.

### 3.5 Sequence alignment

To compare the differences among *Rhynchocypris* species, mitogenome sequences of other 7 *Rhynchocypris* species were downloaded from Genbank and included in this study (Table 4).

**Table 4:** Mitochondrial genome of the *Rhynchocypris* species used in this study

Species	Length (bp)	A + T %	AT‐skew	GC‐skew	Accession number	Reference
*R. oxycephalus*	16,609	56.0	0.030	0.196	MH885043	This study
*R. percnurus*	16,608	55.8	0.034	0.204	KT359599	Unpublished
*R. lagowskii*	16,603	55.7	0.039	0.205	KF734881	Unpublished
*R. perenurus mantschuricus*	16,602	57.9	0.030	0.200	AP009061	Saitoh et al. (2006)
*R. percnurus sachalinensis*	16,599	58.0	0.033	0.214	AP009150	Imoto et al. (2013)
*R. kumgangensis*	16,604	54.5	0.038	0.221	JQ675733	Yun, Yu, Kim, and Kwak (2012)
*R. semotilus*	16,605	55.7	0.025	0.193	KT748874	Yu, Kim, and Kim (2017)
*R. oxycephalus jouyi*	16,607	55.8	0.025	0.191	AB626852	Imoto et al. (2013)

The complete mitochondrial genome of 13 PCGs, tRNA and their combined sequence, rRNA and their combined sequence was all aligned by Clustal X 1.83 (Jeanmougin et al., 1998), and the results are shown in Table 5.

**Table 5:** Some feature of mitochondrial genomes and different regions of 8 *Rhynchocypris* species

	Length of consensus sequence	Amount of variable sites	Kimura 2‐Parameter distance	Ts/Tv ratios	Base composition			
					T	C	A	G
Mitochondrion genome	15,684	3,417 (21.8%)	0.097	4.72	26.9	26.6	28.8	17.6
ND1	975	297 (30.5%)	0.141	4.18	29.8	27.7	25.7	16.8
ND2	1,047	360 (34.4%)	0.163	4.13	26.0	31.1	27.6	15.4
COI	1,551	314 (20.2%)	0.088	5.91	31.1	24.8	25.8	18.3
COII	691	139 (20.1%)	0.086	7.14	28.5	25.2	29.1	17.2
ATP8	165	40 (24.2%)	0.107	7.34	23.9	29.0	34.8	12.3
ATP6	684	181 (26.5%)	0.117	4.09	31.2	26.2	28.1	14.6
COIII	785	150 (19.1%)	0.090	7.01	29.7	26.8	25.5	18.0
ND3	351	93 (26.5%)	0.116	6.32	29.3	28.4	26.0	16.3
ND4L	297	60 (20.2%)	0.086	5.65	28.0	29.9	26.4	15.7
ND4	1,383	395 (28.6%)	0.139	4.69	29.1	27.3	28.0	15.7
ND5	1,839	555 (30.2%)	0.145	4.51	28.8	27.8	28.5	14.9
ND6	522	166 (31.8%)	0.147	6.37	38.3	14.4	16.0	31.2
Cytb	1,141	275 (24.1%)	0.112	3.93	30.2	27.0	26.8	16.0
12S rRNa	957	71 (7.4%)	0.029	4.94	19.5	26.3	30.2	24.0
16S rRNA	1,693	177 (10.5%)	0.040	2.78	21.1	23.1	34.4	21.3
Combined sequences of tRNA genes	2,650	249 (9.4%)	0.036	3.23	20.5	24.3	32.9	22.3
Combined sequences of rRNA genes	1,566	124 (8.0%)	0.028	7.12	26.6	21.1	28.6	23.7

According to Brown, George, and Wilson (1979) and Knight and Mindell (1993) conclusions that the conversion ratio of the gene sequence was lower than 2.0, it was generally considered that the mutation had reached saturation and it was likely to be affected by the evolutionary noise, so special weighting must be carried out to ensure the comparison in the process of constructing the evolutionary relationship of the system with the correct information. It could be found that all of the Ts/Tv ratio was higher than 2.0, which indicated the conversion and transversion were not saturated. And it was suitable for phylogenetic analysis. In addition, It can be found that G content in the most segments was very low, which indicated an obvious antibias in the Guanine.

According to variable sites and the Kimura‐2‐Parameter distance (Table 5), it could be found that ND2 had the maximum mutation rate (34.4%) and genetic distance (0.163) among 13 PCGs, which was in accordance with Qiao's (2014) conclusion. While COII had a small mutation rate and genetic distance, it could be indicated that the sequence was very conservative.

### 3.6 Phylogenetic analysis

**Figure 7:**
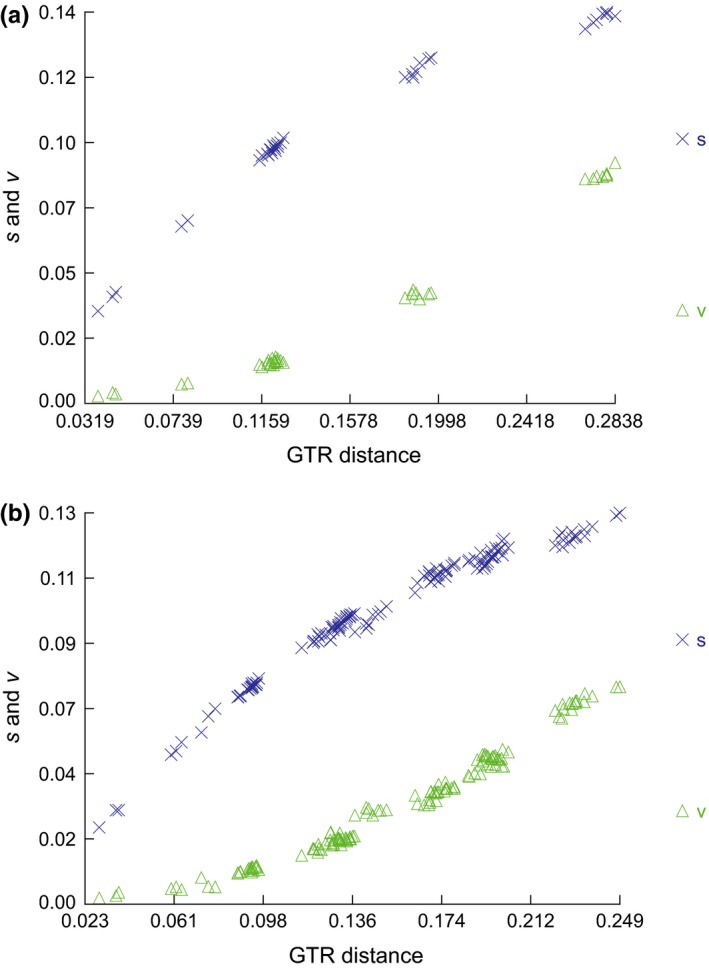
(a) Saturation plot for the substitutions of 13 protein‐coding genes; (b) saturation plot for the substitutions of the complete mitochondrial genome (excepted D‐loop)

**Figure 8:**
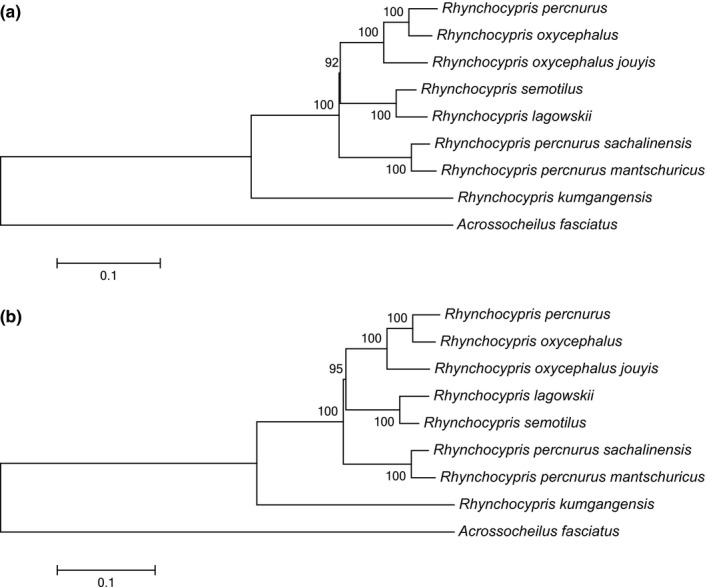
(a) The maximum likelihood analyses of phylogenetic relationship based on 12 PCGs (excepted ND6) of 8 *Rhynchocypris* species. (b) The maximum likelihood analyses of phylogenetic relationship based on 13 PCGs of 8 *Rhynchocypris* species. *Acrossocheilus fasciatus* was selected as outgroup to root the tree in both (a) and (b)

Based on 13 PCGs of 8 *Rhynchocypris* species, we established a phylogenetic tree by maximum likelihood method with 1,000 replications which set *Acrossocheilus fasciatus* as outgroup. Before the phylogenetic analysis, we used software DAMBE (Xia, 2013) to analyze the substitution saturation of PCGs of *Rhynchocypris* species and compared the transition and transversion rate of mitochondrial DNA with GTR distance to verify whether there was mutation saturation in the *Rhynchocypris* species’ PCGs. The results showed that transition and transversion rate were not saturated and could be used for phylogenetic analysis (Figure 7a). In addition, Miya and Nishida (2000a, 2000b) suggested that ND6 gene should be excluded from phylogenetic analysis because of its heterogeneous base composition and consistently poor phylogenetic performance. So we established another phylogenetic tree excluded ND6 gene. The results of two phylogenetic analyses were almost the same. *R. percnurus*, *R. oxycephalus,* and *R. o. jouyi* appeared as sister group to *R. lagowskii* and *R. semotilus*. And the combined group could form a sister group with *R. p. sachalinensis* and *R. p. mantschuricus. R. kumgangensis* had a farther relationship with other *Rhynchocypris* species, but it could cluster with others. Two phylogenetic trees are shown in Figure 8a, b.

To further investigate the phylogenetic relationships of *Leuciscus* species, the phylogenetic relationships were reconstructed based on the complete mitochondrial genome. 17 species including one species of *Pseudaspius*, two species of *Tribolodon*, two species of *Phoxinus*, one species of *Oreoleuciscus,* and two species of *Leuciscus* were used to perform the phylogenetic analysis (Table 6). Because of the fast mutation rate in D‐loop region, this region was excluded from phylogenetic analysis. The maximum likelihood and Bayesian trees were constructed based on the complete mitochondrial genome (except D‐loop), with *Acrossocheilus fasciatus* as outgroup.

**Table 6:** Mitochondrial genome of the *Leuciscus* species used in this study

Genus	Species	Length (bp)	Accession number	Reference
*Pseudaspius*	*P. leptocephalus*	16,604	AP009058	Saitoh et al. (2006)
*Tribolodon*	*T.hakonensis*	16,602	AB626855	Imoto et al. (2013)
	*T. brandtii*	16,598	NC_018819	Imoto et al. (2013)
*Phoxinus*	*P. phoxinus*	17,859	AP009309	Imoto et al. (2013)
	*P. ujmonensis*	17,738	KJ000673	Xu et al. (2013)
*Oreoleuciscus*	*O. potanini*	16,602	AB626851	Imoto et al. (2013)
*Leuciscus*	*L. burdigalensis*	16,607	KT223568	Hinsinger et al. (2015)
	*L. waleckii*	16,605	NC_018825	Wang et al. (2013)
*Acrossocheilus*	*A. fasciatus*	16,589	KF781289	Cheng et al. (2015)

**Figure 9:**
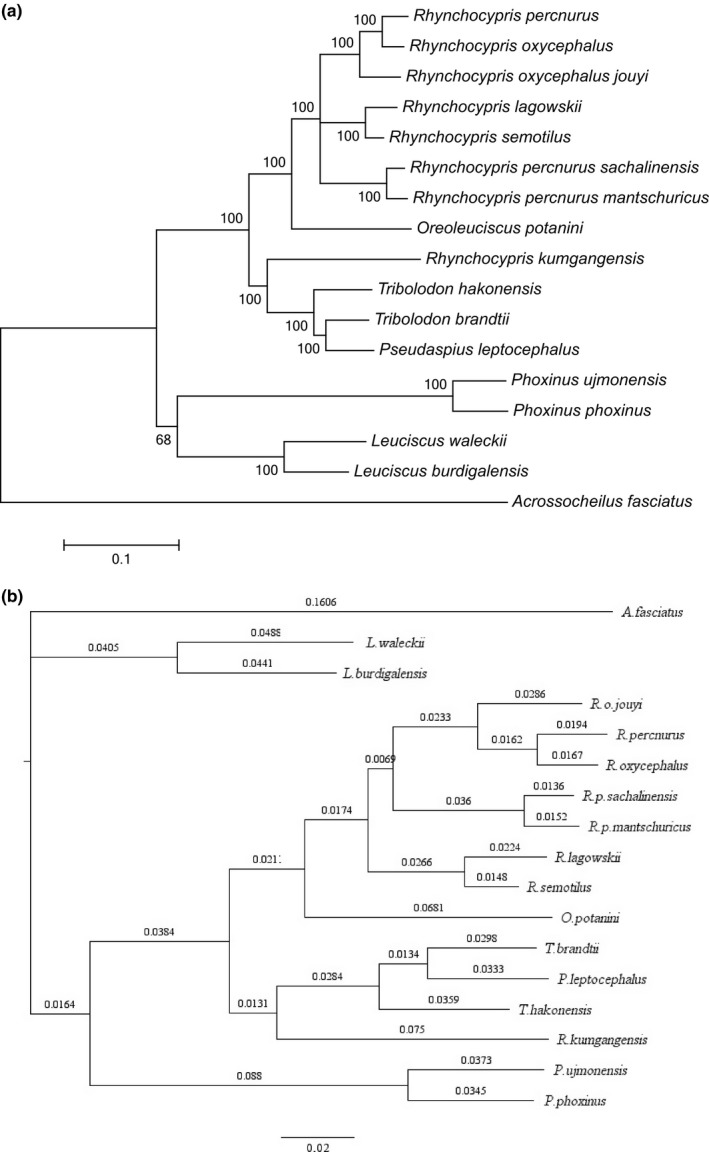
(a) The phylogenetic relationship among 17 *Leuciscus* fishes based on the complete mitochondrial genome (excepted D‐loop) from maximum likelihood analyses. The bootstrap support values are shown above the branches. (b) The phylogenetic relationship among 17 *Leuciscus* species based on the complete mitochondrial genome (excepted D‐loop) from Bayesian analyses. *Acrossocheilus fasciatus* was selected as outgroup to root the tree in both (a) and (b)

The results of substitution saturation showed that transition and transversion rate were not saturated and can be used for phylogenetic analysis (Figure 7b). The topology of the maximum likelihood and Bayesian trees constructed based on the complete sequence of the mitochondrial genome was identical. As the result, *R. oxycephalus*, *R. percnurus*, *R. lagowskii*, *R. p. mantschuricus*, *R. p. sachalinensis*, *R. semotilus, and A. fasciatus* were clustered as a monophyletic group. The group appeared as sister group to *R. kumgangensis*, *Pseudaspius leptocephalus*, *Tribolodon hakonensis,* and *T. brandtii*. The combined monophyly could form a sister group with *Phoxinus phoxinus* and *P. ujmonensis*. And the combined monophyly could form a sister group with *Leuciscus burdigalensis* and *L. waleckii*. Two types of phylogenetic trees are shown in Figure 9a, b.

### 3.7 The analysis of the DNA barcoding

We used the software MEGA 5.0 (Tamura et al., 2011) to calculate the amount of variable sites and variation rate among the 6 PCGs (COI, COIII, Cytb, ND2, ND4, and ND5) in the *Rhynchocypris*. The variation rate of the 6 PCGs was 23.0%, 24.8%, 31.8%, 42.1%, 34.7%, and 35.5%, respectively. The variation rate was relatively large. All of the 6 PCGs were considered as good DNA bar codes in *Rhynchocypris* species.

**Figure 10:**
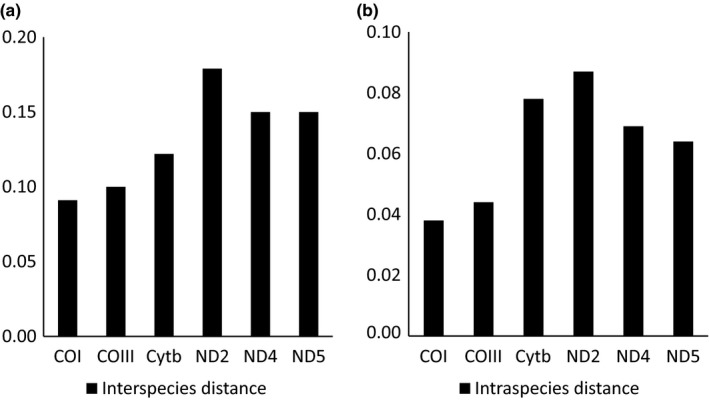
Kimura 2‐parameter distance among 6 selected protein‐coding genes. (a) interspecies (b) intraspecies

The mean interspecies and intraspecies distance used by Kimura‐2‐Parameter model among 6 PCGs is shown in Figure 10. According to Figure 10, we could learn that ND2 had the maximum interspecies distance among 6 PCGs, while COI had the minimum. And the interspecies distance of ND2 was obviously larger than other 5 PCGs. The relationship among 6 PCGs is COI < COIII < Cytb < ND4 = ND5 < ND2. In addition, ND2 had the maximum intraspecies distance among 6 PCGs, while COI had the minimum. And the intraspecies distance of COI and COIII was obviously larger than other 4 PCGs. The relationship among 6 PCGs is COI < COIII < ND4 < ND5 < Cytb < ND2.

According to the degree of differentiation of genetic variation, software SPSS 19.0 was used to perform the Wilcoxon test on 6 PCGs (Tables 6, 7a, b). We compared the test values of different segments, as the basis for segment filtering. By the significance level of *p* < 0.05, the result of the Wilcoxon test in interspecies distance in *Rhynchocypris* species was COI < COIII < Cytb < ND4 = ND5 < ND2. And the result of the Wilcoxon test in intraspecies distance in *Rhynchocypris* species was COI = COIII < ND5 < ND4 = Cytb <= ND2. The results were basically consistent with the results of the sequence alignment.

**Table 7:** (a) The Wilcoxon test of interspecific divergence among *Rhynchocypris* species; (b) The Wilcoxon test of intraspecific divergence among *Rhynchocypris* species

W^+^	W^−^	Relative ranks, *n*, *p* value	Result
(a)			
COI	COIII	W^+^ = 6,698, W^−^ = 19,408, *n* = 228, *p* < 0.001	COI < COIII
COI	ND2	W^+^ = 0, W^−^ = 26,106, *n* = 228, *p* < 0.001	COI < ND2
COI	ND4	W^+^ = 0, W^−^ = 26,106, *n* = 228, *p* < 0.001	COI < ND4
COI	ND5	W^+^ = 0, W^−^ = 26,106, *n* = 228, *p*<0.001	COI < ND5
COI	Cytb	W^+^ = 399, W^−^ = 25,707, *n* = 228, *p* < 0.001	COI < Cytb
COIII	ND2	W^+^ = 141, W^−^ = 25,965, *n* = 228, *p* < 0.001	COIII < ND2
COIII	ND4	W^+^ = 607, W^−^ = 25,499, *n* = 228, *p* < 0.001	COIII < ND4
COIII	ND5	W^+^ = 722, W^−^ = 25,384, *n* = 228, *p* < 0.001	COIII < ND5
COIII	Cytb	W^+^ = 5,162, W^−^ = 20,944, *n* = 228, *p* < 0.001	COIII < Cytb
ND2	ND4	W^+^ = 25,965, W^−^ = 141, *n* = 228, *p* < 0.001	ND2 > ND4
ND2	ND5	W^+^ = 25,808, W^−^ = 198, *n* = 228, *p* < 0.001	ND2 > ND5
ND2	Cytb	W^+^ = 24,235, W^−^ = 1871, *n* = 228, *p* < 0.001	ND2 > Cytb
ND4	ND5	W^+^ = 14,286, W^−^ = 11,820, *n* = 228, *p* = 0.216	ND4 = ND5
ND4	Cytb	W^+^ = 3,852, W^−^ = 22,254, *n* = 228, *p* < 0.001	ND4 > Cytb
ND5	Cytb	W^+^ = 3,827, W^−^ = 22,279, *n* = 228, *p* < 0.001	ND5 > Cytb
(b)			
COI	COIII	W^+^ = 48, W^−^ = 142, *n* = 25, *p* = 0.058	COI = COIII
COI	ND2	W^+^ = 0, W^−^ = 153, *n* = 25, *p* < 0.001	COI < ND2
COI	ND4	W^+^ = 0, W^−^ = 153, *n* = 25, *p* < 0.001	COI < ND4
COI	ND5	W^+^ = 0, W^−^ = 153, *n* = 25, P = *p*<0.001	COI < ND5
COI	Cytb	W^+^ = 10, W^−^ = 180, *n* = 25, *p* = 0.001	COI < Cytb
COIII	ND2	W^+^ = 22, W^−^ = 168, *n* = 25, *p* = 0.003	COIII < ND2
COIII	ND4	W^+^ = 34, W^−^ = 156, *n* = 25, *p* = 0.014	COIII < ND4
COIII	ND5	W^+^ = 37, W^−^ = 153, *n* = 25, *p* = 0.020	COIII < ND5
COIII	Cytb	W^+^ = 26, W^−^ = 184, *n* = 25, *p* = 0.003	COIII < Cytb
ND2	ND4	W^+^ = 150, W^−^ = 3, *n* = 25, *p* < 0.001	ND2 > ND4
ND2	ND5	W^+^ = 150, W^−^ = 3, *n* = 25, *p* < 0.001	ND2 > ND5
ND2	Cytb	W^+^ = 112, W^−^ = 78, *n* = 25, *p* = 0.494	ND2 = Cytb
ND4	ND5	W^+^ = 150, W^−^ = 34, *n* = 25, *p* = 0.044	ND4 > ND5
ND4	Cytb	W^+^ = 72, W^−^ = 118, *n* = 25, *p* = 0.355	ND4 = Cytb
ND5	Cytb	W^+^ = 53, W^−^ = 137, *n* = 25, *p* = 0.091	ND5 = Cytb

**Figure 11:**
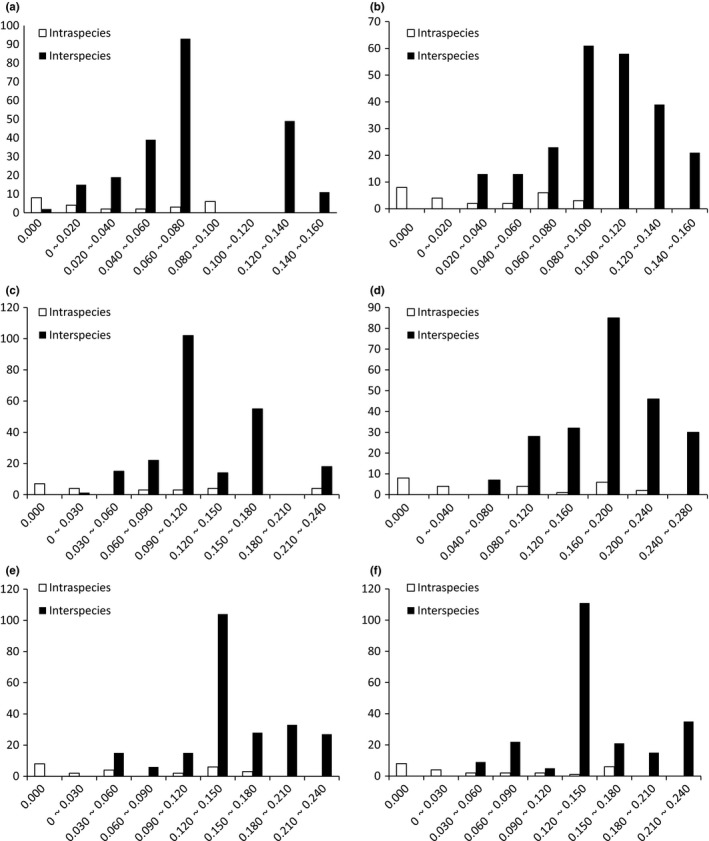
Distribution of interspecific and intraspecific variations of *Rhynchocypris* species. (a) COI sequence; (b) COIII sequence; (c) Cytb sequence; (d) ND2 sequence; (e) ND4 sequence; (f) ND5 sequence

According to the theory of the ideal DNA barcoding by Meyer and Paulay (2005), the interspecies variation of the ideal DNA barcoding should be significantly larger than the intraspecies variation, and there should be a gap between the two, which called DNA barcoding gap. Distribution of interspecific and intraspecific variations of *Rhynchocypris* species in 6 PCGs is shown in Figure 11. We found that the average interspecies distance between 6 PCGs was larger than the intraspecies distance, and there were different degrees of overlap between intraspecies and interspecies distribution of each PCG. All 6 PCGs had no obvious DNA barcoding gap. However, COI, Cytb, and ND2 genes had less overlap between intraspecies and interspecies distribution which was beneficial to species differentiation.

## 4 DISCUSSION

### 4.1 Structural features of the mitochondrial genome of R. oxycephalus

In this study, the complete sequence of the mitochondrial genome of *R. oxycephalus* was obtained. *R. oxycephalus* had the same characteristics as other Cyprinidae species in mitochondrial genome structures, with a total length of 16,609 bp and a mitochondrial genome A + T content of 56.0% which was consistent with the A + T preference of vertebrates. It indicated that the order of mitochondrial genomes changes rarely, and it was suitable for solving the biological system developmental relationship of higher order elements such as families and subjects (Boore, 1999). Base G had the lowest content in the mitochondrial genome of *R. oxycephalus*. The phenomenon might be related to the way the mitochondrial gene is replicated. Specifically, the H chain replicated first, and when the H chain replication reached the origin of light‐strand replication, the L chain began to replicate. It caused a relatively long L chain in a single‐stranded state was prone to base mutations, resulting in a more stable G base being gradually replaced by other bases (Clayton, 1982). There were several intergenic regions and overlapping regions in the mitochondrial genome, including 12 intergenic regions and seven overlapping regions. This phenomenon was also common in other Cyprinidae species (Wu et al., 2009; Zhang et al., 2009).

Among 13 PCGs of *R. oxycephalus*, like other vertebrates, except ND6 gene, all genes showed strong A + T bias and C base preference. ND6 gene was the PCG of the L chain, so it could be indicated that there were large base composition differences between the genes encoded by the H chain and the L chain. *R. oxycephalus*'s PCGs start codon was relatively constant and had the general characteristics of bony fish (Chang, Huang, & Lo, 1994), while the stop codon changed greatly. Beside complete stop codons, there were two types of incomplete stop codons (T/TA). This phenomenon was widespread in the mitochondrial genome. It was not difficult to see the transcript of these protein sequences was U or UA at the 3' end. Due to the Ploy A at the 3' end of the mRNA, a complete stop codon could be formed by the addition of polyadenylation during processing (Ojala, Montoya, & Attardi, 1981). Among 22 tRNA genes of *R. oxycephalus*, in addition to tRNA‐Ser (AGY), the rest could fold into a typical clover structure. The tRNA‐Ser (AGY) lacked the DHU arm and formed a single‐loop structure at the position of the DHU arm. This structure was very common in fish mitochondrion (Lee & Kocher, 1995; Noack, Zardoya, & Meyer, 1996). Cheng et al.(2015) had shown that this tRNA lacking the DHU arm could adjust the structural morphology and it did not affect its ability to enter the ribosome and its ability to carry and transport amino acids. In addition, the putative origin of light‐strand replication was a region with a fast rate of evolution and a high degree of variation, which could fold into a stable stem‐loop secondary structure. Similar structures were found in fishes, amphibians, and mammals, but not in reptiles and birds (Ojala et al., 1981; Wolstenholme, 1992). Generally speaking, the control region of mitochondrial genome played an important role in regulating gene replication and transcription. On the other hand, the sequence length of the control region was also closely related to the length of the whole mitochondrial genome. The control region consisted of termination‐associated sequence, central conserved domain, and conserved sequence block. Termination‐associated sequence was the most variable part of the control region, which is involved in termination of DNA replication (Hai, Yang, Wei, Ming, & Hu, 2003). In termination‐associated sequence, there was an obvious hairpin structure (TACAT and ATGTA). Several TACAT sequences could also be found in downstream sequence (Lin et al., 2006). Central conserved domain was the most conservative zone in the control zone, and it was very conservative in almost all fishes. It could identify three conserved regions including CSB‐D, CSB‐E, and CSB‐F by comparing with other Cyprinidae species. Conserved sequence block could identify three conserved regions including CSB1, CSB2, and CSB3. It was presumed that this region was involved in the occurrence of H chain RNA primers (Walberg & Clayton, 1981). CBS2 and CBS3 were generally conservative in fish, but CBS1 varied greatly (Liu, Wu, Zhu, & Zhuang, 2010). The result of Tandem Repeats Finder analysis showed that there had an AT repetitive area among 816–847 bp in the control region. The sequence AT repeated 15 times. This area was also found in other Cyprinidae species (Liu, Tzeng, & Teng, 2002). Different repetition times of AT sequence resulted in different length of conservative sequence region of fish.

### 4.2 The phylogenetic relationships of Rhynchocypris species

In recent years, more and more researches on genus *Rhynchocypris* were presented. Imoto et al. (2013) considered that genus *Tribolodon*, *Pseudaspius*, *Rhynchocypris* from East Asia, and genus *Oreoleuciscus* are clustered as a monophyly. And this monophyly can form a sister group with genus *Phoxinus*. Xu (2013) combined mitochondrial genes (16SrRNA, Ctyb) with nuclear genes (Rag1, Rag2), analyzed three problems including: (a) whether *Rhynchocypris* species form a monophyletic group; (b) the phylogenetic position of genus *Rhynchocypris*; and (c) the intrageneric phylogeny of genus *Rhynchocypris*. Xu (2013) concluded that genus *Rhynchocypris* is a polyphyletic group and its phylogenetic position should be redefined.

In this study, the maximum likelihood and Bayesian analyses were performed based on the complete mitochondrial genome and 13 PCGs of *Rhynchocypris* and *Leuciscus* species, and the topological structure of these two trees based on complete sequence of mitochondrial genome was identical. All the trees had high bootstrap supporting values. The result indicated that genus *Rhynchocypris* is a polyphyletic group and *R. kumgangensis* had distant relationship with other *Rhynchocypris* species. This conclusion was consistent with former results (Imoto et al., 2013; Xu, 2013). However, the phylogenetic position of *R. p. sachalinensis* and *R. p. mantschuricus* was different from Imoto's analysis (2013) which clustered them with *R. percnurus*. The possible reasons for these results might be the geographical difference of the selected fish and the different genes used for alignment. It showed that the phylogenetic relationship of certain species in *Rhynchocypris* is still not very clear. More genome sequences and more different *Rhynchocypris* species from different regions should be used for phylogenetic analysis to determine the relationship in *Rhynchocypris* species.

In general, although a few consistent results were obtained, due to the small amount of samples, the phylogenetic relationship of *Rhynchocypris* species remains to be further analyzed and validated based on a wider range of species and more sequences combined with numerous analytical tools.

### 4.3 DNA barcoding of Rhynchocypris species

Nowadays, more and more people use different mitochondrial genes as DNA bar codes to identify animal species. By establishing a phylogenetic tree for 13 PCGs, Tang, Zheng, Ma, Cheng, and Li (2017) concluded ND5 gene had the potential to be DNA bar code for Octopodidae. The validation results generally in accordance with the traditional morphological classification. By analyzing the SNP loci, Sperling, Rosengarten, Moreno, and Dellaporta (2012) concluded that ND2 and ND5 genes can be used as a supplement of COI gene for DNA barcoding. In addition, Chen, Jiang, and Qiao (2012) used three gene sequences of COI, COII, and Cytb to verify the possibility of DNA barcoding technology in the identification of insect germplasm and proposed “TAG,” which is used as germplasm identification according to different TAG. The control region can also be barcoded by the TAG method though it is the hypervariable region of mitochondrion (Chen et al., 2012).

In theory, the ideal DNA barcoding sequence should have large variation between species, small intraspecific variation, and DNA barcoding gap. In this study, the interspecies distance of the 6 PCGs we selected is all larger than the intraspecies distance. Relatively speaking, COI and ND2 genes have larger interspecies distance and smaller intraspecies distance. So, the effect of using these two PCGs to analyze the genetic distance is better than the other four PCGs. In addition, we can find the DNA barcoding gap in six PCGs. Moritz and Cicero (2004) suggested that if there are many closely related species in the collected samples, the overlap between the interspecies variation and the intraspecies variation will increase, so the DNA barcode gap may not exist. Another reason may be that there may be hybridization or genetic introgression between these species in the neighborhood, which will increase the overlap between interspecific and intraspecific variations. The phenomenon is also present in other *Rhynchocypris* species (Xu, 2013). Relatively speaking, COI, Cytb, and ND2 genes had less overlap between intraspecies and interspecies distribution. So, we concluded that COI and ND2 genes are suitable DNA bar codes for *Rhynchocypris* species. For different subjects, it is necessary to compare different DNA barcoding genes. In view of the fact that the published sequence of COI gene of *Rhynchocypris* species in GenBank is more than ND2 gene and COI has perfect universal primers, COI is more convenient and efficient to conduct research. So we recommend using COI sequence as the DNA bar code for the identification of *Rhynchocypris* species and ND2 gene can be used for assisted identification.

## CONFLICT OF INTEREST

None declared.

## AUTHORS CONTRIBUTION

QC conceived the ideas and designed the study; QC, ZZ, and YG performed the experiments and collected the data; ZZ and QC analyzed the data; QC, ZZ, and YG interpreted the results; ZZ and QC wrote the manuscript. All authors contributed critically to the drafts and gave final approval for publication.

## Data Availability

All data used in this study are publicly available in NCBI databases (GenBank accession nos.: MH885043, KJ000673, KT223568, KT359599, KT748874, KF734881, KF781289, AB626851, AB626852, AB626855, AP009058, AP009061, AP009150, AP009309, JQ675733, NC_018819, NC_018825).

## References

[ece35369-bib-0001] Anderson, S. , Bankier, A. T. , Barrell, B. G. , de Bruijn, M. H. , Coulson, A. R. , Drouin, J. , … Young, I. G. (1981). Sequence and organization of the human mitochondrial genome. Nature, 290(5806), 457–465. 7219534 10.1038/290457a0

[ece35369-bib-0002] Benson, G. (1999). Tandem repeats finder: A program to analyze DNA sequences. Nucleic Acids Research, 27(2), 573–580. 10.1093/nar/27.2.573 9862982 PMC148217

[ece35369-bib-0003] Boore, J. L. (1999). Survey and summary, animal mitochondrial genomes. Nucleic Acids Research, 27(8), 1767–1780. 10101183 10.1093/nar/27.8.1767 PMC148383

[ece35369-bib-0004] Bozdogan, H. (1987). Model selection and Akaike's Information Criterion (AIC): The general theory and its analytical extensions. Psychometrika, 52(3), 345–370. 10.1007/BF02294361

[ece35369-bib-0005] Brown, W. M. , George, M. , & Wilson, A. C. (1979). Rapid evolution of animal mitochondrial DNA. Proceedings of the National Academy of Sciences of the USA, 76(4), 1967–1971. 10.1073/pnas.76.4.1967 109836 PMC383514

[ece35369-bib-0006] Castresana, J. (2000). Selection of conserved blocks from multiple alignments for their use in phylogenetic analysis. Molecular Biology & Evolution, 17(4), 540–552. 10.1093/oxfordjournals.molbev.a026334 10742046

[ece35369-bib-0007] Chang, Y. S. , Huang, F. L. , & Lo, T. B. (1994). The complete nucleotide sequence and gene organization of carp (*Cyprinus carpio*) mitochondrial genome. Journal of Molecular Evolution, 38(2), 138–155. 10.1007/BF00166161 8169959

[ece35369-bib-0008] Chen, R. , Jiang, L. Y. , & Qiao, G. X. (2012). The effectiveness of three regions in mitochondrial genome for aphid DNA barcoding: A case in Lachininae. PLoS ONE, 7(10), e46190 10.1371/journal.pone.0046190 23056258 PMC3463548

[ece35369-bib-0009] Chen, S. J. , Chi, C. B. , Mu, Y. L. , Liu, W. D. , & Zhou, Z. C. (2008). Informative efficiencies of mitochondrial genes in phylogenetic analysis of teleostean. Journal of Fishery Sciences of China, 276(5317), 1318–1319.

[ece35369-bib-0010] Cheng, S. H. , Yan, J. J. , Liu, Y. L. , Lu, Y. M. , Zhang, Y. , Xia, M. N. , & Yan, Y. Z. (2015). The complete mitochondrial genome of the *Acrossocheilus fasciatus* (Cyprinidae, Barbinae). Mitochondrial DNA, 26(6), 941–942. 24409932 10.3109/19401736.2013.863300

[ece35369-bib-0011] Clayton, D. A. (1982). Replication of animal mitochondrial DNA. Cell, 28(4), 693–705. 10.1016/0092-8674(82)90049-6 6178513

[ece35369-bib-0012] Drummond, A. J. , Buxton, S. , Cheung, M. , Cooper, A. , Heled, J. , Kearse, M. , … Wilson, A. (2010). Geneious Version 4.8.5. Software Available. http://www.geneious.com/

[ece35369-bib-0013] Field, A. (2013). Discovering Statistics using IBM SPSS Statistics[M]. London, UK: Sage Publications Ltd.

[ece35369-bib-0014] Guo, X. , Liu, S. , & Liu, Y. (2003). Comparative analysis of the mitochondrial DNA control region in cyprinids with different ploidy level. Aquaculture, 224(1), 25–38. 10.1016/S0044-8486(03)00168-6

[ece35369-bib-0015] Gutell, R. R. , Lee, J. C. , & Cannone, J. J. (2002). The accuracy of ribosomal RNA comparative structure models. Current Opinion in Structural Biology, 12(3), 301–310. 10.1016/S0959-440X(02)00339-1 12127448

[ece35369-bib-0016] Hai, L. , Yang, G. , Wei, F. , Ming, L. , & Hu, J. (2003). Sequence variability of the mitochondrial DNA control region and population genetic structure of sika deers (*Cervus nippon*) in China. Acta Zoologica Sinica, 49(1), 53–60.

[ece35369-bib-0017] Hajibabaei, M. , Janzen, D. H. , Burns, J. M. , Hallwachs, W. , & Hebert, P. D. (2006). DNA barcodes distinguish species of tropical Lepidoptera. Proceedings of the National Academy of Sciences of the United States of America, 103(4), 968–971. 10.1073/pnas.0510466103 16418261 PMC1327734

[ece35369-bib-0018] Hebert, P. D. N. , Ratnasingham, S. , & Dewaard, J. R. (2003). Barcoding animal life: Cytochrome c oxidase subunit 1 divergences among closely related species. Proceedings of the Royal Society of London. Series B: Biological Sciences, 270, S96. 12952648 10.1098/rsbl.2003.0025 PMC1698023

[ece35369-bib-0019] Hinsinger, D. D. , Debruyne, R. , Thomas, M. , Denys, G. P. J. , Menesson, M. , Utge, J. , & Dettai, A. (2015). Fishing for barcodes in the Torrent: From COI to complete mitochondrial genome on NGS platforms. DNA Barcodes (Berlin), 3, 170–186.

[ece35369-bib-0020] Hogg, I. D. , & Hebert, P. D. N. (2004). Biological identification of springtails (Hexapoda: Collembola) from the Canadian Arctic, using mitochondrial DNA barcodes. Canadian Journal of Zoology, 82(6), 749–754. 10.1139/z04-041

[ece35369-bib-0021] Huelsenbeck, J. P. , & Ronquist, F. (2005). Bayesian analysis of molecular evolution using MrBayes In Nielsen R. (Ed.), Statistical methods in molecular evolution (pp. 183–226). New York, NY: Springer.

[ece35369-bib-0022] Imoto, J. M. , Saitoh, K. , Sasaki, T. , Yonezawa, T. , Adachi, J. , Kartavtsev, Y. P. , … Hanzawa, N. (2013). Phylogeny and biogeography of highly diverged freshwater fish species (Leuciscinae, Cyprinidae, Teleostei) inferred from mitochondrial genome analysis. Gene, 514(2), 112–124. 10.1016/j.gene.2012.10.019 23174367

[ece35369-bib-0023] Ito, Y. , Sakai, H. , Shedko, S. , & Jeon, S. R. (2002). Genetic differentiation of the northern Far East *Cyprinids*, *Phoxinus* and *Rhynchocypris* . Fisheries Science, 68, 75–78.

[ece35369-bib-0024] Jeanmougin, F. , Thompson, J. D. , Gouy, M. , Higgins, D. G. , & Gibson, T. J. (1998). Multiple sequence alignment with Clustal X. Trends in Biochemical Sciences, 23(10), 403–405. 10.1016/S0968-0004(98)01285-7 9810230

[ece35369-bib-0025] Kawaguchi, A. , Miya, M. , & Nishida, M. (2001). Complete mitochondrial DNA sequence of *Aulopus japonicus* (Teleostei: Aulopiformes), a basal Eurypterygii: Longer DNA sequences and higher‐level relationships. Ichthyological Research, 48(3), 213–223. 10.1007/s10228-001-8139-0

[ece35369-bib-0026] Knight, A. , & Mindell, D. P. (1993). Substitution bias, weighting of DNA sequence evolution, and the phylogenetic position of Fea's Viper. Systematic Biology, 42(1), 18–31. 10.1093/sysbio/42.1.18

[ece35369-bib-0027] Lalitha, S. (2000). Primer premier 5. Biotech Software & Internet Report, 1(6), 270–272. 10.1089/152791600459894

[ece35369-bib-0028] Lee, W. J. , & Kocher, T. D. (1995). Complete sequence of a sea lamprey (*Petromyzon Marinus*) mitochondrial genome: Early establishment of the vertebrate genome organization. Genetics, 139(2), 873. 7713438 10.1093/genetics/139.2.873 PMC1206387

[ece35369-bib-0029] Li, X. R. , Liu, S. F. , Li, D. , Du, T. F. , & Zhuang, Z. M. (2015). Species identification and phylogenetic relationships in order *Clupeiformes* based on DNA barcoding. Journal of Fishery Sciences of China, 22(6), 1133–1141.

[ece35369-bib-0030] Liang, Y. , Sui, X. , Chen, Y. , Jia, Y. , & He, D. (2014). Life history traits of the Chinese minnow *Rhynchocypris oxycephalus* in the upper branch of Yangtze River, China. Zoological Studies, 53(1), 1–10. 10.1186/s40555-014-0036-0

[ece35369-bib-0031] Lin, G. , Lo, L. C. , Zhu, Z. Y. , Feng, F. , Chou, R. , & Yue, G. H. (2006). The Complete Mitochondrial genome sequence and characterization of single‐nucleotide polymorphisms in the control region of the Asian Seabass (*Lates calcarifer*). Marine Biotechnology, 8(1), 71–79. 10.1007/s10126-005-5051-z 16228120 PMC4273291

[ece35369-bib-0032] Liu, H. Z. (2002). Structure and evolution of the mitochondrial DNA control region of fish: Take Rhodeinae for instance. Progress in Natural Science, 12(3), 266–270.

[ece35369-bib-0033] Liu, H. Z. , Tzeng, C. S. , & Teng, H. Y. (2002). Sequence variations in the mitochondrial DNA control region and their implications for the phylogeny of the Cypriniformes. Canadian Journal of Zoology, 80(3), 569–581. 10.1139/z02-035

[ece35369-bib-0034] Liu, S. F. , Wu, R. X. , Zhu, L. , & Zhuang, Z. M. (2010). Complete sequence and gene organization of mitochondrial dna of the small yellow croaker *Larimichthys polyactis* . Oceanologia Et Limnologia Sinica, 4(2), 347–351.

[ece35369-bib-0035] Lohse, M. , Drechsel, O. , Kahlau, S. , & Bock, R. (2013). Organellar Genome DRAW‐ a suite of tools for generating physical maps of plastid and mitochondrial genomes and visualizing expression data sets. Nucleic Acids Research, 41, 575 10.1093/nar/gkt289 23609545 PMC3692101

[ece35369-bib-0036] Lowe, T. M. , & Chan, P. P. (2016). tRNAscan‐SE on‐line: Search and contextual analysis of transfer RNA genes. Nucleic Acids Research, 44, 54–57. 10.1093/nar/gkw413 PMC4987944 27174935

[ece35369-bib-0037] Mayfield, J. E. , & Mckenna, J. F. (1978). A‐T rich sequences in vertebrate DNA. A possible explanation of q‐banding in metaphase chromosomes. Chromosoma, 67(2), 157 10.1007/BF00293173 567568

[ece35369-bib-0038] Meyer, C. P. , & Paulay, G. (2005). DNA barcoding: Error rates based on comprehensive sampling. PLOS Biology, 3(12), e422 10.1371/journal.pbio.0030422 16336051 PMC1287506

[ece35369-bib-0039] Miya, M. , & Nishida, M. (2000a). Molecular systematics of the deep‐sea fish genus *Gonostoma* (Stomiiformes: Gonostomatidae): Two paraphyletic clades and resurrection of *Sigmops* . Copeia, 2000(2), 378–389.

[ece35369-bib-0040] Miya, M. , & Nishida, M. (2000b). Use of mitogenomic information in teleostean molecular phylogenetics: A tree‐based exploration under the maximum‐parsimony optimality criterion. Molecular Phylogenetics & Evolution, 17(3), 437–455. 10.1006/mpev.2000.0839 11133198

[ece35369-bib-0041] Miya, M. , Sato, Y. , Fukunaga, T. , Sado, T. , Poulsen, J. Y. , Sato, K. , … Iwasaki, W. (2015). MiFish, a set of universal PCR primers for metabarcoding environmental DNA from fishes: detection of more than 230 subtropical marine species. Royal Society Open Science, 2(7), 150088. 26587265 10.1098/rsos.150088 PMC4632578

[ece35369-bib-0042] Moritz, C. , & Cicero, C. (2004). DNA barcoding: Promise and pitfalls. Plos Biology, 2(10), e354 10.1371/journal.pbio.0020354 15486587 PMC519004

[ece35369-bib-0043] Nelson , G. , & Joseph , S. (1976). Fishes of the world. Quarterly Review of Biology, 7(1995), 1–16.

[ece35369-bib-0044] Noack, K. , Zardoya, R. , & Meyer, A. (1996). The complete mitochondrial DNA sequence of the bichir (*Polypterus ornatipinnis*), a basal ray‐finned fish: Ancient establishment of the consensus vertebrate gene order. Genetics, 144(3), 1165–1180. 8913758 10.1093/genetics/144.3.1165 PMC1207609

[ece35369-bib-0045] Ojala, D. , Montoya, J. , & Attardi, G. (1981). tRNA punctuation model of RNA processing in human mitochondria. Nature, 290(5806), 470–474. 7219536 10.1038/290470a0

[ece35369-bib-0046] Park, I. S. , Im, J. H. , Ryu, D. K. , Nam, Y. K. , & Dong, S. K. (2010). Effect of starvation on morphometric changes in *Rhynchocypris oxycephalus* (sauvage and dabry). Journal of Applied Ichthyology, 17(6), 5.

[ece35369-bib-0047] Perna, N. T. , & Kocher, T. D. (1995). Patterns of nucleotide composition at fourfold degenerate sites of animal mitochondrial genomes. Journal of Molecular Evolution, 41(3), 353–358. 10.1007/BF01215182 7563121

[ece35369-bib-0048] Posada, D. , & Crandall, K. A. (1998). MODELTEST: Testing the model of DNA substitution. Bioinformatics, 14(9), 817–818. 10.1093/bioinformatics/14.9.817 9918953

[ece35369-bib-0049] Qiao, H. Y. (2014). Phylogeny of Schizothoracinae fishes and genetic structure of Pampus argenteus population based on mitochondrial DNA sequences. Shanghai, China: Shanghai Ocean University.

[ece35369-bib-0050] Reuter, J. S. , & Mathews, D. H. (2010). RNAstructure: Software for RNA secondary structure prediction and analysis. BMC Bioinformatics, 11(1), 873 10.1186/1471-2105-11-129 PMC2984261 20230624

[ece35369-bib-0051] Saitoh, K. , Hayashizaki, K. , Yokoyama, Y. , Asahida, T. , Toyohara, H. , & Yamashita, Y. (2000). Complete nucleotide sequence of Japanese flounder (*Paralichthys olivaceus*) mitochondrial genome: Structural properties and cue for resolving teleostean relationships. Journal of Heredity, 91(4), 271 10.1093/jhered/91.4.271 10912672

[ece35369-bib-0052] Saitoh, K. , Sado, T. , Mayden, R. L. , Hanzawa, N. , Nakamura, K. , Nishida, M. , & Miya, M. (2006). Mitogenomic evolution and interrelationships of the Cypriniformes (Actinopterygii: Ostariophysi): The first evidence toward resolution of higher‐level relationships of the world's largest freshwater fish clade based on 59 whole mitogenome sequences. Journal of Molecular Evolution, 63(6), 826 10.1007/s00239-005-0293-y 17086453

[ece35369-bib-0053] Sasaki, T. , Kartavtsev, Y. P. , Chiba, S. N. , Uematsu, T. , Sviridov, V. V. , & Hanzawa, N. (2007). Genetic divergence and phylogenetic independence of Far Eastern species in subfamily *Leuciscinae* (Pisces: Cyprinidae) inferred from mitochondrial DNA analyses. Genes & Genetic Systems, 82(4), 329 10.1266/ggs.82.329 17895584

[ece35369-bib-0054] Simon, C. , Frati, F. , Beckenbach, A. , Crespi, B. , Liu, H. , & Floor, P. (1994). Evolution, weighting, and phylogenetic utility of mitochondrial gene sequences and a compilation of conserved polymerase chain reaction primers. Annals of the Entomological Society of America, 87, 651–701. 10.1093/aesa/87.6.651

[ece35369-bib-0055] Sperling, E. A. , Rosengarten, R. D. , Moreno, M. A. , & Dellaporta, S. L. (2012). The complete mitochondrial genome of the verongid sponge *Aplysina cauliformis*: Implications for DNA barcoding in demosponges. Hydrobiologia, 687(1), 61–69. 10.1007/s10750-011-0879-x

[ece35369-bib-0056] Sui, X. , Liang, Y. , & He, D. (2016). The complete mitochondrial genome of *Rhynchocypris oxycephalus* (Cypriniformes: Cyprinidae). DNA Sequence, 27(5), 3. 10.3109/19401736.2015.1018224 25799350

[ece35369-bib-0057] Sun, Q. , Wang, D. , & Wei, Q. (2016). The complete mitochondrial genome of *Phoxinus lagowskii* (Teleostei, Cypriniformes: Cyprinidae). DNA Sequence, 27(2), 2. 10.3109/19401736.2014.919462 24865913

[ece35369-bib-0058] Tamura, K. , Peterson, D. , Peterson, N. , Glen, S. , Masatoshi, N. , & Sudhir, K. (2011). MEGA5: Molecular evolutionary genetics analysis using maximum likelihood, evolutionary distance, and maximum parsimony methods. Molecular Biology & Evolution, 28(10), 2731–2739. 10.1093/molbev/msr121 21546353 PMC3203626

[ece35369-bib-0059] Tang, Y. , Zheng, X. , Ma, Y. , Cheng, R. , & Li, Q. (2017). The complete mitochondrial genome of *Amphioctopus marginatus* (Cephalopoda: Octopodidae) and the exploration for the optimal DNA barcoding in Octopodidae. Conservation Genetics Resources, 10(1), 1–4.

[ece35369-bib-0060] Walberg, M. W. , & Clayton, D. A. (1981). Sequence and properties of the human KB cell and mouse L cell D‐loop regions of mitochondrial DNA. Nucleic Acids Research, 9(20), 5411–5421. 10.1093/nar/9.20.5411 7301592 PMC327529

[ece35369-bib-0061] Wang, B. , Ji, P. , Xu, J. , Sun, J. , Yang, J. , Xu, P. , & Sun, X. (2013). Complete mitochondrial genome of *Leuciscus waleckii* (Cypriniformes: Cyprinidae: Leuciscus). Mitochondrial DNA, 24(2), 126–128. 23098409 10.3109/19401736.2012.731406

[ece35369-bib-0062] Ward, R. D. , Zemlak, T. S. , Innes, B. H. , Last, P. R. , & Hebert, P. D. (2005). DNA barcoding Australia's fish species. Philosophical Transactions of the Royal Society of London. Series B, Biological Sciences, 360(1462), 1847–1857. 10.1098/rstb.2005.1716 16214743 PMC1609232

[ece35369-bib-0063] Wolstenholme, D. R. (1992). Animal mitochondrial DNA: Structure and evolution. International Review of Cytology, 141(6), 173. 1452431 10.1016/s0074-7696(08)62066-5

[ece35369-bib-0064] Wu, X. , Wang, L. , Chen, S. , Zan, R. , Xiao, H. , & Zhang, Y. (2009). The complete mitochondrial genomes of two species from *Sinocyclocheilus* (Cypriniformes: Cyprinidae) and a phylogenetic analysis within Cyprininae. Molecular Biology Reports, 37(5), 2163–2171. 10.1007/s11033-009-9689-x 19688279

[ece35369-bib-0065] Xia, X. (2013). DAMBE5: A comprehensive software package for data analysis in molecular biology and evolution. Molecular Biology & Evolution, 30(7), 1720–1728. 10.1093/molbev/mst064 23564938 PMC3684854

[ece35369-bib-0067] Xu, W. (2013). Studies on molecular phylogeny of *Rhynchocypris* fishes (Teleostei: Cyprinidae) and phylogeography of cold‐adapting freshwater fishes in the eastern. Shanghai, China: Fudan University.

[ece35369-bib-0068] Yoo, H. S. , Eah, J. Y. , Kim, J. S. , Kim, Y. J. , Min, M. S. , Paek, W. K. , … Kim, C. B. (2013). Retraction note: DNA barcoding Korean birds. Molecules & Cells, 35(4), 357–357. 10.1007/s10059-013-3151-6 23568538 PMC3887896

[ece35369-bib-0069] Yu, J. N. , Kim, B. J. , & Kim, C. (2017). The complete mitochondrial genome of the black star fat minnow (*Rhynchocypris semotilus*), an endemic and endangered fish of Korea. Mitochondrial DNA Part A, 28(1), 114–115. 10.3109/19401736.2015.1111348 26709416

[ece35369-bib-0070] Yun, Y. E. , Yu, J. N. , Kim, S. , & Kwak, M. (2012). The complete mitochondrial genome of Kumgang fat minnow *Rhynchocypris kumgangensis* (Cypriniformes, Leuciscinae) in Korea. Mitochondrial DNA, 23(5), 347. 22708852 10.3109/19401736.2012.690752

[ece35369-bib-0071] Zardoya, R. , & Meyer, A. (1996). Phylogenetic performance of mitochondrial protein‐coding genes in resolving relationships among vertebrates. Molecular Biology and Evolution, 13(7), 933–942. 10.1093/oxfordjournals.molbev.a025661 8752002

[ece35369-bib-0072] Zhang, C. G. , Zhao, Y. H. , Xing, Y. C. , Guo, R. L. , Feng, Y. , Zhang, Q. , & Fan, E. Y. (2011). Fish species diversity and conservation in Beijing and adjacent areas. Biodiversity Science, 19(5), 597–604.

[ece35369-bib-0073] Zhang, X. , Yue, B. , Jiang, W. , & Song, Z. (2009). The complete mitochondrial genome of rock carp *Procypris rabaudi* (Cypriniformes: Cyprinidae) and phylogenetic implications. Molecular Biology Reports, 36(5), 981 10.1007/s11033-008-9271-y 18496768

